# Supplementary material for: Physalis alkekengi L. Calyx Extract Alleviates Glycolipid Metabolic Disturbance and Inflammation by Modulating Gut Microbiota, Fecal Metabolites, and Glycolipid Metabolism Gene Expression in Obese Mice
Source: Nutrients. 2023 May 28;15(11):2507. doi: 10.3390/nu15112507 (PMC10255802; doi:10.3390/nu15112507)
Supplement: Supplementary file 1 [file nutrients-15-02507-s001.zip › nutrients-2410538-supplementary.pdf]

Untargeted Metabolomics Analysis

For metabolomics analysis, the liquid-mass system consisted of the Waters Acquity I-Class PLUS ultra-high performance liquid chromatography tandem with the Waters Xevo G2-XS QTOF high-resolution mass spectrometer and Acquity UPLC HSS T3 column (1.8 μm 2.1*100 mm), purchased from Waters (Milford, USA). For the positive ion mode (POS), mobile phase A consisted of 0.1% formic acid aqueous solution, and mobile phase B consisted of 0.1% acetonitrile formate. For the negative ion mode (NEG), mobile phase A consisted of 0.1% formic acid aqueous solution, and mobile phase B consisted of 0.1% acetonitrile formate. The injection volume was 1 μL. The original data collected by MassLynx V4.2 were used for the identification and alignment of peaks as well as other data processing operations using Progenesis QI software. The identification was performed based on the online METLIN database of Progenesis QI software and a self-built database of Beijing BioMarker Technology Co., Ltd. (Beijing, China). The theoretical fragment identification was performed at the same time. Parent ion mass number deviation was 100 ppm, and fragment ion mass number was deviation within 50 ppm.

Supplemental Tables

Supplementary Table S1. Sequences of primers used for qPCR in this study.

Name	Sequence（5'-3')
ACC1	F: TTGTTCAGCCGGATCTTGTC<br>R: ACTTCCCGACCAAGGACTTTG
FAS	F: TGAATCAGCCCCACGCAGT<br>R: CCGAGTCAGTCTTGGAGGACAT
PPARα	F: GAGGCAGATGACCTGGAAAGT<br>R: TGCGTGAACTCCGTAGTGGTA
Acox1	F: GTTCTCACGATGCCAATGC<br>R: ATGCTGGGGTTACAGGTTTG
SREBP-1c	F: CACTTCTGGAGACATCGCAAAC<br>R: GTCCTCCTGTGTACTTGCCCA
SCD1	F: TCTTCCTTATCATTGCCAACACCA<br>R: GCGTTGAGCACCAGAGTGTATCG
Cpt1α	F: AGGACCCTGAGGCATCTATT<br>R: ATGACCTCCTGGCATTCTCC
ChREBPα	F: CGACACTCACCCACCTCTTC<br>R: TTGTTCAGCCGGATCTTGTC
G6Pase	F: AGAGACTGTGGGCATCAATCT<br>R: CCGGAATCCATACGTTGATT
PEPCK	F: GCCGACCTCCCTTAGAAATAG<br>R: CGAACTTCGGAGAACAGACGTGA
MCP-1	F: TCTGGACCCATTCCTTCTTG<br>R: TCTGGACCCATTCCTTCTTG
IL-6	F: CAAGAAAGACAAAGCCAGAGTCCTT<br>R: CAAGAAAGACAAAGCCAGAGTCCTT
TNFα	F: GCCACCACGCTCTTCTGCCT<br>R: GCCACCACGCTCTTCTGCCT
IL − 1β	F: CCGTGATGATGACCTGAGGAG<br>R: CAAGACAGGTATAGATTCTTGTC

Supplementary Figure

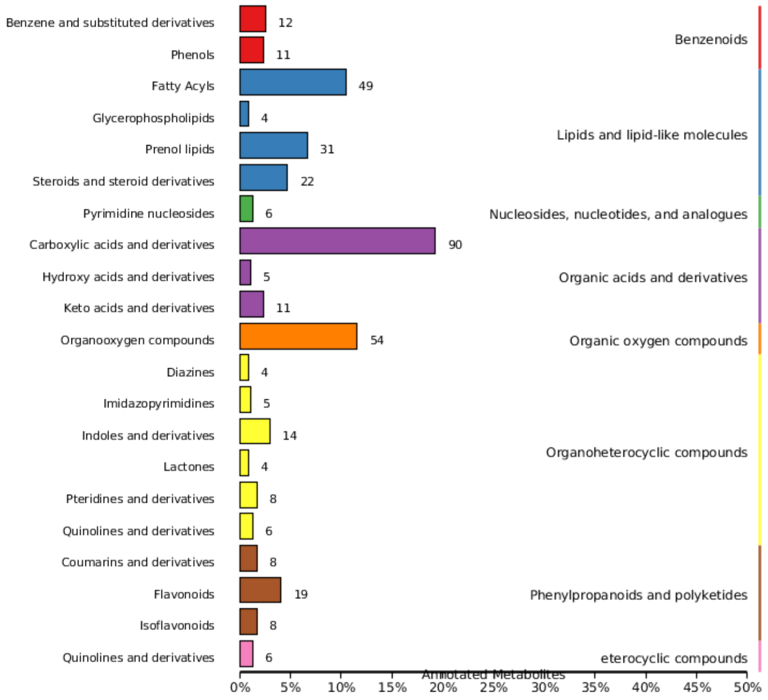

Supplementary Figure S1. The number of metabolites were annotated and classified based on the HMDB database of PC extract.

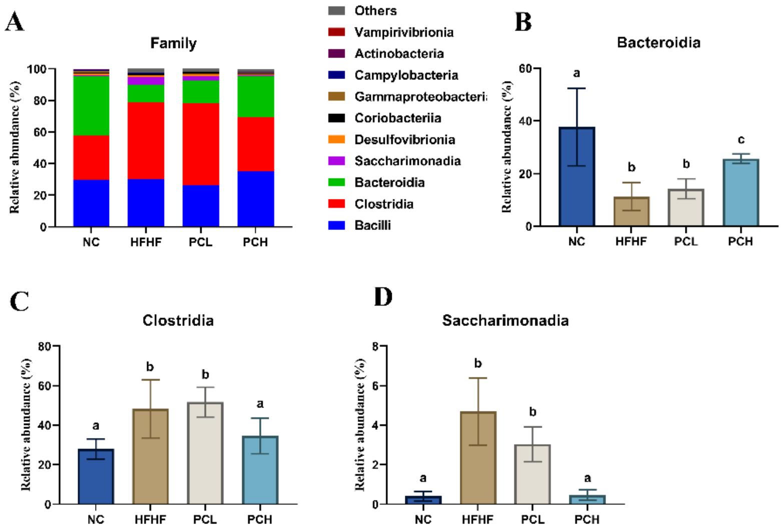

Supplementary Figure S2. Bacterial taxonomic profiling in the family level of gut microbiota at individual level, (E-H) significantly changes ($p < 0.05$) of the composition of the gut microbiota at family taxa level. Data are expressed as mean ± SD, Bars marked with different superscript letters (a–c) indicate significant differences at $p < 0.05$.

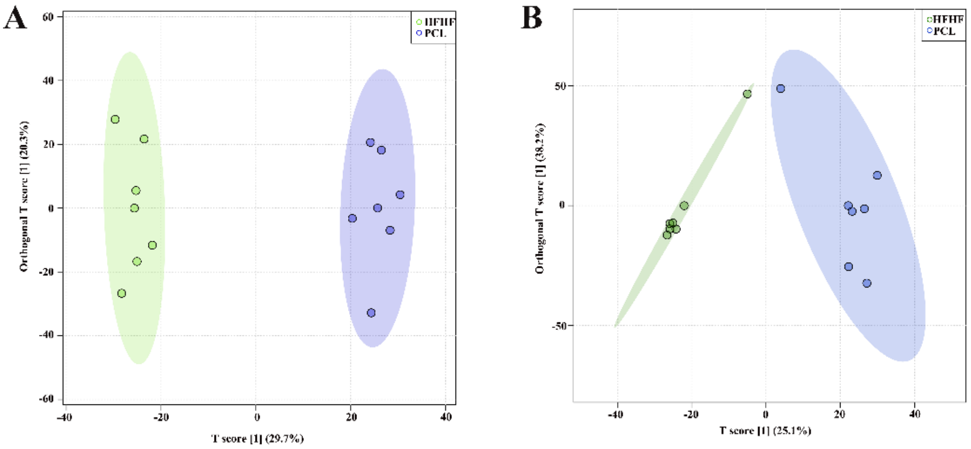

Supplementary Figure 3. Multivariate statistical analysis of fecal metabolites measured by untargeted metabolomics analysis at (**A**) positive and (**B**) negative ion mode.

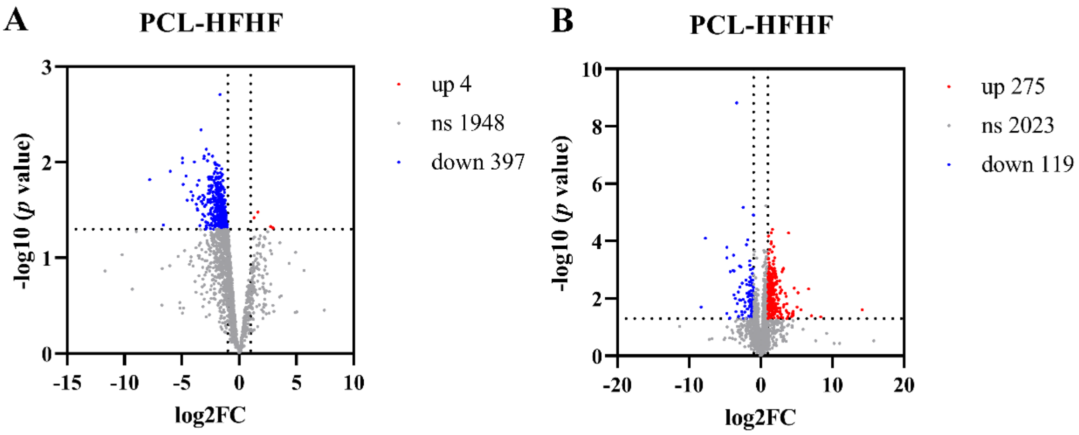

Supplementary Figure S4. Expression of differential metabolites in the PCL and HFHF groups was represented by Volcano plot (**A**) at positive and (**B**) negative ion mode.

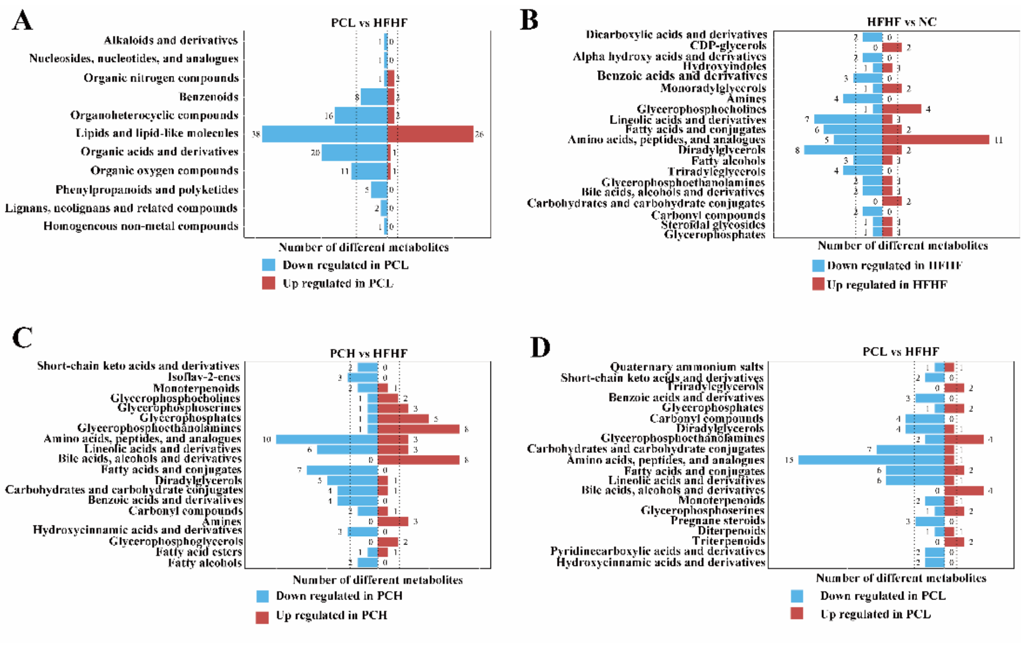

Supplementary Figure S5. (**A**) The number of differential metabolites were annotated and classified based on the HMDB database changed by PCL, (**B**–**D**) the differential metabolites were annotated and classified at subclasses in NC, PCH, and PCL group compared with the HFHF group.

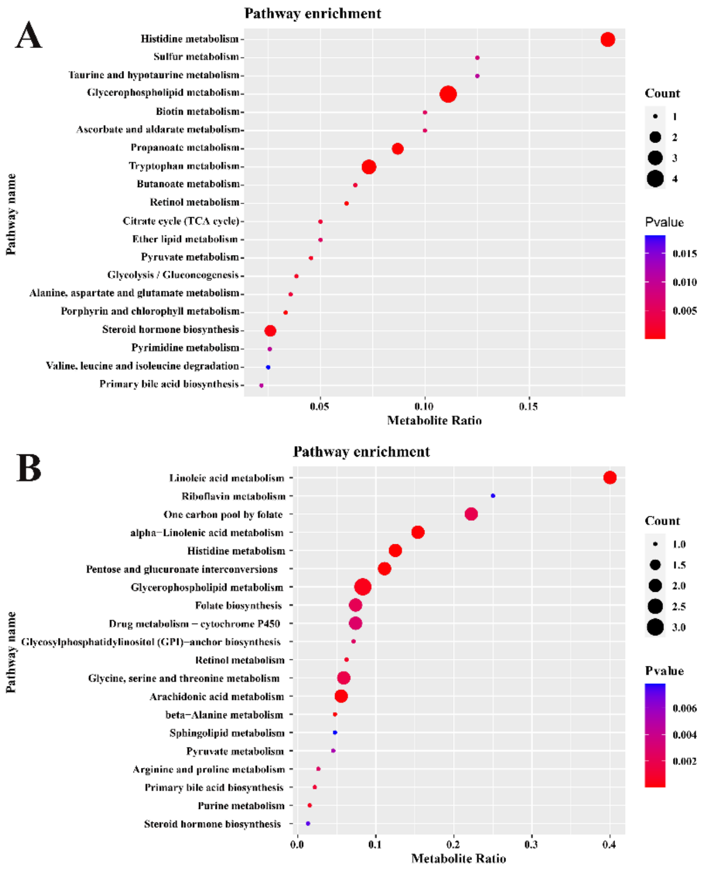

Supplementary Figure S6. The enrichment pathway of fecal differential metabolites by Kyoto encyclopedia of genes and genomes (KEGG) analysis.

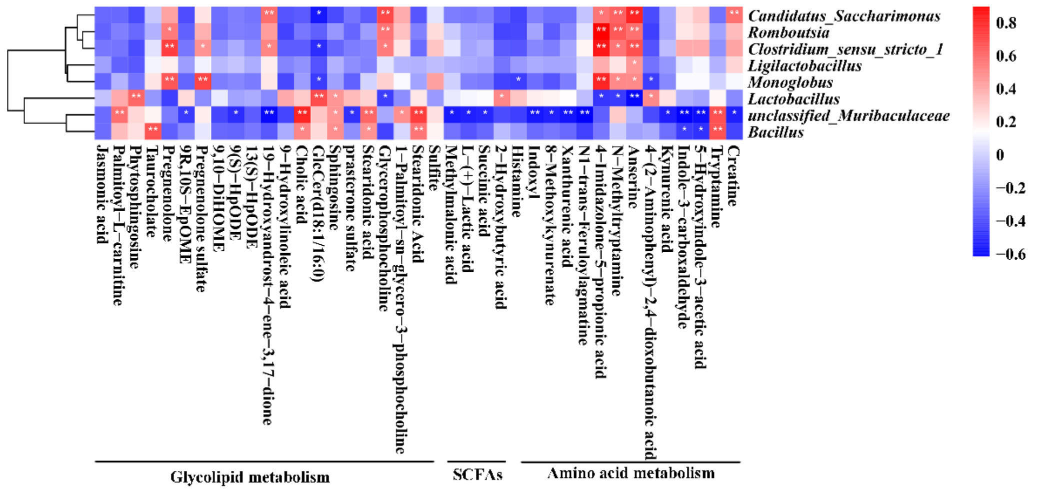

Supplementary Figure S7. Two-factor correlation network analysis showing correlations among significant difference specific gut microbiota, co-regulated metabolites in feces.
